# A Thermostable Glucoamylase from *Bispora* sp. MEY-1 with Stability over a Broad pH Range and Significant Starch Hydrolysis Capacity

**DOI:** 10.1371/journal.pone.0113581

**Published:** 2014-11-21

**Authors:** Huifang Hua, Huiying Luo, Yingguo Bai, Kun Wang, Canfang Niu, Huoqing Huang, Pengjun Shi, Caihong Wang, Peilong Yang, Bin Yao

**Affiliations:** 1 Key Laboratory for Feed Biotechnology of the Ministry of Agriculture, Feed Research Institute, Chinese Academy of Agricultural Sciences, Beijing, 100081, P. R. China; 2 CAAS-ICRAF Joint Laboratory on Agroforestry and Sustainable Animal Husbandry, Beijing, 100193, P. R. China; Virginia Tech, United States of America

## Abstract

**Background:**

Glucoamylase is an exo-type enzyme that converts starch completely into glucose from the non-reducing ends. To meet the industrial requirements for starch processing, a glucoamylase with excellent thermostability, raw-starch degradation ability and high glucose yield is much needed. In the present study we selected the excellent Carbohydrate-Activity Enzyme (CAZyme) producer, *Bispora* sp. MEY-1, as the microbial source for glucoamylase gene exploitation.

**Methodology/Principal Findings:**

A glucoamylase gene (*gla15*) was cloned from *Bispora* sp. MEY-1 and successfully expressed in *Pichia pastoris* with a high yield of 34.1 U/ml. Deduced GLA15 exhibits the highest identity of 64.2% to the glucoamylase from *Talaromyces* (*Rasamsonia*) *emersonii*. Purified recombinant GLA15 was thermophilic and showed the maximum activity at 70°C. The enzyme was stable over a broad pH range (2.2–11.0) and at high temperature up to 70°C. It hydrolyzed the breakages of both α-1,4- and α-1,6-glycosidic linkages in amylopectin, soluble starch, amylose, and maltooligosaccharides, and had capacity to degrade raw starch. TLC and H^1^-NMR analysis showed that GLA15 is a typical glucoamylase of GH family 15 that releases glucose units from the non-reducing ends of α-glucans. The combination of *Bacillus licheniformis* amylase and GLA15 hydrolyzed 96.14% of gelatinized maize starch after 6 h incubation, which was about 9% higher than that of the combination with a commercial glucoamylase from *Aspergillus niger*.

**Conclusion/Significance:**

GLA15 has a broad pH stability range, high-temperature thermostability, high starch hydrolysis capacity and high expression yield. In comparison with the commercial glucoamylase from *A. niger*, GLA15 represents a better candidate for application in the food industry including production of glucose, glucose syrups, and high-fructose corn syrups.

## Introduction

Starch is a polysaccharide carbohydrate of plant cells, and is composed solely of α-glucose units that are linked by α-1,4- or α-1,6-glycosidic bonds. It has two homopolysaccharide types: amylopectin and amylose. Amylopectin is an α-1,4-linked d-glucose polymer with approximately 5% of α-1,6-linked branches, whereas amylose is a linear polymer essentially consisting of α-1,4-linked glucopyranose residues [1]. Starch is one of the most important renewable natural resources and is used in many industries including textile, laundry, pharmaceutics, paper making and food manufacture. In food industry, starch is mainly used to produce glucose, which can be further utilized to produce high-fructose syrups, bioethanol, organic acids and amino acids [2],[3].

Mass production of glucose from starch is a two-stage process, involving α-amylase and glucoamylase. In the first step, starch slurry is gelatinized, followed by liquefaction rapidly by a thermostable α-amylase at 95–105°C and pH 6.0–6.5 [4]. In the following saccharification step, the obtained dextrin is further hydrolyzed to β-d-glucose by glucoamylase. Currently, *Aspergillus niger* glucoamylase is widely used in industry. It has the temperature and pH optima at 60–65°C and 4.0–4.5, respectively, and retains only 10% of initial activity after incubation at 70°C for 30 min [5]. Thus adjustment of temperature and pH from liquefaction to saccharification process is necessary, which not only requires additional equipments but also causes higher production cost.

Great efforts have been attempted to improve the performance of glucoamylase, including screening of glucoamylase-producing thermotolerant and thermophilic organisms [5], site-directed mutagenesis [6], and directed evolution [7],[8]. Unfortunately, these works only made limited contribution to the improvement of economical industrial glucoamylases. Exploration of novel glucoamylases is another strategy. Glucoamylases from thermoacidophilic and thermophilic bacteria have been reported, including *Clostridium thermohydrosulfuricum* [9], *Clostridium thermosaccharolyticum* [10], and *Thermoanaerobacter tengcongensis* MB4 [11]. In comparison with fungal glucoamylases, these enzymes are active and stable at high temperatures (60–70°C), but have low production yields. Archaea growing at temperatures higher than 60°C is another promising microbial source of biocatalysts for industrial starch processing. Some glucoamylases from hyperthermophilic *Sulfolobus* [12], *Thermoplasma* [13], *Picrophilus* and *Methanococcus* [14] have been purified and characterized. But there are still many obstacles to be overcome, such as cultivation of hyperthermophilic archaea and poor heterogeneous expression. Thermophilic fungi represent an ideal source for thermostable glucoamylases with high yield. So far several thermophilic glucoamylases have been identified in *Talaromyces* [15], *Thermomyces* [16], and *Chaetomium* [17]. These glucoamylases usually show extremely high temperature optima and thermostability, but the glucose yield from starch degradation is quite low [18]. Thus a glucoamylase with excellent thermostability, raw-starch degradation ability and high glucose yield is still needed to meet the industrial requirements for high temperature starch processing.

*Bispora* sp. MEY-1 is an excellent Carbohydrate-Activity Enzyme (CAZyme) producer [19]–[25]. In this study, we cloned a glucoamylase gene from *Bispora* sp. MEY-1 and expressed the gene product in *Pichia pastoris* at high level. The recombinant glucoamylase (GLA15) showed better thermostability and pH stability than all other fungal counterparts reported before, and had wide substrate specificity and great hydrolysis capacity.

## Materials and Methods

### Strains, media, vectors and chemicals

*Bispora* sp. MEY-1 (CGMCC 2500; the China General Microbiological Culture Collection Center, Beijing, China) was cultivated in wheat bran medium [19] at 30°C for 3–5 days to induce the production of glucoamylase as described previously [22]. *Escherichia coli* Trans1-T1 and the plasmid pEASY-T3 from TransGen (Beijing, China) were used for gene cloning and sequencing. *Pichia pastoris* GS115 and plasmid pPIC9 (Invitrogen, Carlsbad, CA, USA) were used for gene expression. *P. pastoris* expression media were prepared as described in the *Pichia* expression kit manual (Invitrogen).

Maltooligosaccharides, 4-nitrophenyl-α-d-maltopentaoside (*p*-NPG5), amylose, amylopectin, glycogen, soluble starch, dextran and isomaltose were purchased from Sigma-Aldrich (St. Louis, MO, USA). The Genome walking kit and restriction endonucleases were from TaKaRa (Otsu, Japan). T4 DNA ligase and endoglycosidase H (Endo H) were from New England Biolabs (Hitchin, UK). All other chemicals were of analytical grade and commercially available.

### Cloning of the glucoamylase gene (*gla15*) from *Bispora* sp. MEY-1

Based on the conserved motifs WGRPQRDG and YDAV(I/L)YQW of glucoamylases of GH 15, a degenerate primer set Gamy-F (5′-TGGGGHCGTCCDCARMGNGAYGG-3′) and Gamy-R (5′-CCACTGRTARAYNGCRTCRTA-3′) was designed and used to clone the core region of glucoamylase gene. The PCR products were cloned into the vector pEASY-T3 and sequenced. Based on the known sequences, six specific primers were designed to obtain the 5′ and 3′ flanking regions (data not shown). The gene fragments were assembled and subject to sequence analysis.

Total RNA was isolated from the mycelia of *Bispora* sp. MEY-1 using the total RNA isolation system kit (Promega, Madison, WI, USA) according to the manufacture's instructions. Reverse transcription was carried out with the First Strand cDNA Synthesis Kit (TOYOBO, Osaka, Japan). The full-length cDNA of the glucoamylase gene was amplified by PCR primers GAΙF (5′-ATGCAAGTATCAACTTGCCTATTTGCGCTATG-3′) and GAΙR (5′-TTACTGCCAACTATCATTCACCTCAGCAACCCC-3′). The PCR product was cloned into vector pEASY-T3 and sequenced.

### Heterologous expression of *gla15* in *P. pastoris*

The gene fragment coding for the mature protein without the signal peptide sequence was amplified by PCR with the expression primers GamyF 5′-CTAGAATTCGCTCCAAGGCAGGAAAATCTGTTACGGCG-3′ and GamyR 5′-GACGCGGCCGCTTACTGCCAACTATCATTCACCTCAGCAACCCC-3′ (*Eco*RΙ and *Not*Ι restriction sites underlined, respectively). The PCR products were digested with *Eco*RΙ and *Not*I, isolated by gel electrophoresis, purified, and cloned into *Eco*RΙ/*Not*I digested pPIC9 to construct the recombinant plasmid pPIC9-*gla15*. The recombinant plasmid was linearized with *Bgl*II and transformed into *P. pastoris* GS115 competent cells by electroporation. Positive transformants were screened based on the glucoamylase activities as described below. The positive transformant with highest glucoamylase activity was selected for further fermentation in 1 l conical flasks containing 400 mL of BMGY at 30°C with an agitation rate of 250 rpm for 48 h. Cells were harvested by centrifugation at 5,000× *g*, 4°C for 10 min and resuspended in 200 mL of BMMY containing 0.5% (v/v) methanol for induction at 30°C. Culture samples were taken every 24 h for enzyme activity assay.

### Protein purification

Fermentation broth was collected by centrifugation (4500× *g*, 4°C, 10 min), and then concentrated by ultrafiltration using the vivaflow 50 ultrafiltration membrane with a molecular weight cut-off of 10 kDa (Vivascience, Hannover, Germany). The supernatant was loaded onto a HiTrap Desalting column pre-equilibrated with buffer A (20 mM Na_2_HPO_4_-citric acid buffer, pH 6.5). The crude enzyme was collected and applied to a HiTrap SP XL column pre-equilibrated with buffer A. Proteins were eluted with a linear gradient of 0–0.7 M NaCl in the same buffer. Fractions containing glucoamylase activity were collected and confirmed by SDS-PAGE.

### Enzyme activity assay

Glucoamylase activity was determined with maltose as the substrate. The reaction mixture contained 140 µl of substrate solution [2% (wt/vol) maltose in H_2_O], 130 µl of 100 mM sodium acetate buffer (pH 4.0), and 10 µl of appropriately diluted enzyme. After incubation at 70°C for 10 min, the reaction was terminated by boiling at 100°C for 5 min. The mixture was then centrifuged at 12,000× *g*, 4°C for 2 min, and 140 µl of the supernatant was combined with 2.1 ml of a commercial reagent consisting of glucose oxidase and catalase (Zhongshengbeikong, Beijing, China) at 37°C for 30 min to dye. The absorption was measured at 520 nm, and the enzyme activity was calculated using glucose as a standard. One unit of glucoamylase activity was defined as the amount of enzyme that liberates 1 µmol of glucose per min under assay conditions (pH 4.0, 70°C, 10 min).

### Biochemical characterization

The pH-GLA15 activity profile was determined at 70°C in 100 mM Na_2_HPO_4_-citric acid buffer (pH 2.0–6.0) as described above. The pH stability of recombinant GLA15 was studied by first incubation at 37°C in 100 mM buffers of various pH values (Na_2_HPO_4_-citric acid for pH 2.2–8.0, Tris-HCl for pH 8.0–9.0 and glycine-NaOH for pH 9.0–12.0) for 1 h followed by enzyme activity assay as described above (pH 4.0, 70°C, 10 min).

The temperature-GLA15 activity profile was determined at temperatures ranging from 30 to 90°C in 100 mM of sodium acetate buffer (pH 4.0) for 10 min. For thermostability analysis, the purified recombinant GLA15 was diluted in 100 mM of sodium acetate buffer (pH 4.0) and incubated at 70°C, 75°C and 80°C, respectively. Samples were withdrawn at various intervals (0, 2, 5, 10, 20, 30, and 60 min), and subject to residual glucoamylase activity assay (pH 4.0, 70°C, 10 min).

Kinetic parameters *K*_m_ and *V*_max_ of GLA15 were determined with soluble starch and maltose as substrates. The hydrolysis reactions were performed in 100 mM of sodium acetate buffer containing 0.5–5.0 mg/ml soluble starch or 1.0–10.0 mg/ml maltose at pH 4.0 and 70°C for 5 min. The *K*_m_ and *V*_max_ values were plotted according to the Lineweaver-Burk method.

### Effects of metal ions and other reagents on GLA15 activity

The effects of different metal ions and reagents on glucoamylase activity of GLA15 were evaluated in the reaction mixture containing various metal ions and chemical reagents (Na^+^, K^+^, Li^+^, Ag^+^, Cu^2+^, Mg^2+^, Mn^2+^, Ca^2+^, Ni^2+^, Pb^2+^, Co^2+^, Zn^2+^, Hg^2+^, Fe^3+^, Cr^3+^, SDS and β-mercaptoethanol) at a final concentration of 5 mM and 10 mM. The enzyme activity was measured, and the enzyme activity without any addition was treated as the control.

### Substrate specificity

The substrate specificity of GLA15 was determined by measuring the hydrolysis products of various α-glucans (amylose, amylopectin, glycogen, soluble starch, raw starch, dextran, isomaltose and maltose to maltoheptaose). The reaction mixture consisting of 150 µl of 100 mM sodium acetate (pH 4.0), 0.5% (wt/vol) substrate and 10 µl of enzyme (13.37 U/ml) was incubated at 60°C with agitation of 100 rpm for different intervals. The reaction was stopped by 5 min boiling. After cooling down to room temperature, the mixtures were centrifuged at 12,000× *g* for 5 min and further concentrated through the Ultrafree-CL HV Centrifugal Filter (Millipore, Billerica, MA, USA) with centrifugation at 5000× *g* for 30 min. Aliquots of 1 µl was diluted with 999 µl sterile water and applied to the HPAEC analysis.

### Hydrolysis product analysis

Aliquots of *p*-NPG5 [600 µl of 0.5% (wt/vol) in 100 mM sodium acetate, pH 4.0] and 0.10 U of GLA15 were incubated at 60°C for various durations. Samples were collected, boiled for 5 min to terminate the reaction, and spotted on the TLC plate (Silica gel 60F_254_, Merck, Darmstadt, Germany). *n*-butanol, acetic acid and water at the ratio of 2∶1∶1 (vol/vol/vol) was used as the solvent. The plate was air dried and soaked in ethanol containing 5% concentrated H_2_SO_4_. The plate was then dried and placed in an oven at 110°C for 5 min.

To identify the anomeric configuration of the hydrolysis product by GLA15 hydrolysis, 300 µl of 1% (wt/vol) maltotriose was incubated with 0.13 U of GLA15 for different intervals. The ^1^H-NMR spectrum was observed with a Bruker AVANCE DRX-500 NMR spectrophotometer (Bruker, Fällanden, Switzerland).

### Starch hydrolysis by the combination of amylase and glucoamylase

Complete hydrolysis of starch involves liquefaction and saccharification steps, which are catalyzed by α-amylase and glucoamylase, respectively [26]. The reaction systems (100 ml) containing 10 g of maize starch in 100 mM McIlvaine buffer (pH 6.0) were boiled to gel, followed by addition of 50 U commercial α-amylase from *Bacillus licheniformis* (Longda Co., Linyi, China) and incubation at 90°C for 2 h with agitation (150 rpm). Then the pH was adjusted to 4.0, and the mixture was further incubated at 70°C with addition of 10 U of GLA15 for 2, 4, 6 and 8 h. The commercial glucoamylase CGA from *A. niger* (Longda) was treated as the control at its temperature optimum (60°C). Each experiment had triplicate, and the glucose content was determined as described above. The saccharification efficiency of GLA15 (%) was calculated as the amount of glucose produced×100/the amount of starch.

### Reverse reaction

Glucose solution (30% wt/vol) in 100 mM sodium acetate buffer (pH 4.0) was incubated with various amounts of GLA15 (7.2, 14.4, and 28.8 U/ml) at 60°C for 150 h with agitation of 100 rpm. The reverse reaction products were analyzed using high performance anion exchange chromatography-pulsed amperometric detector (HPAEC-PAD). The samples were eluted in 100 mM NaOH for 15 min.

### Nucleotide sequence accession number

The nucleotide sequence for the cDNA of *Bispora* sp. MEY-1 glucoamylase gene (*gla15*) was deposited in the GenBank database under accession number KJ866875.

## Results

### Gene cloning from *Bispora* sp. MEY-1

A gene fragment of 704 bp was cloned from the genome of *Bispora* sp. MEY-1 with degenerate primers Gamy-F and Gamy-R. The full-length 2001-bp *gla15* was obtained by TAIL-PCR and sequence assembly. The cDNA of *gla15* was then obtained via RT-PCR. The cDNA of *gla15* was 1827 bp in length, and encoded 608 amino acids. SignalP (http://www.cbs.dtu.dk/services/SignalP/) [27] analysis indicated the presence of a putative N-terminal signal peptide at residues 1–16. The theoretical molecular mass of the mature protein was 63.3 kDa.

**Figure 1:**
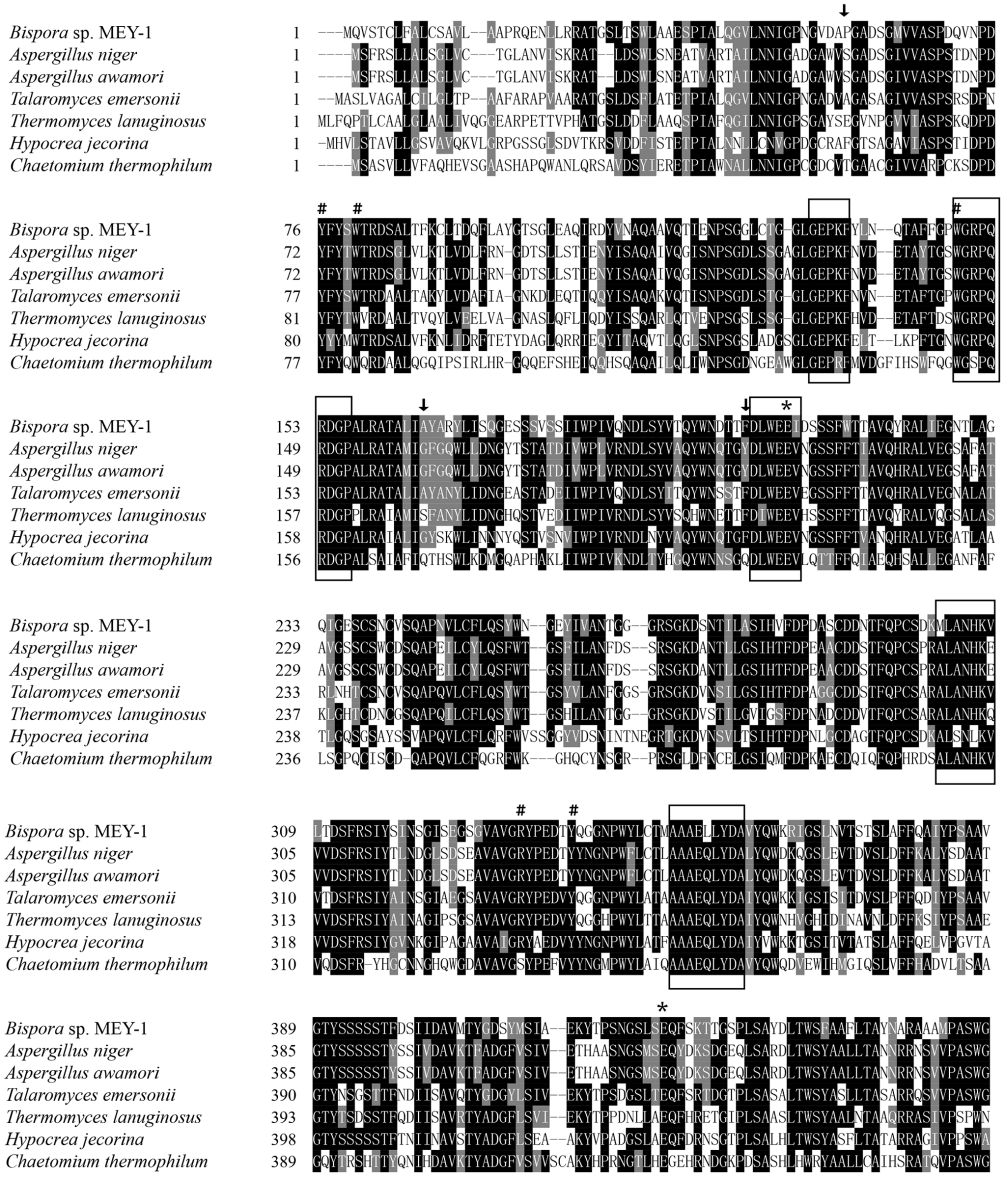
Multiple amino acid sequence alignment of deduced GLA15 with GH15 glucoamylases from *Talaromyces emersonii* (gi: 12666724), *Thermomyces lanuginosus* (gi: 61658242), *Aspergillus niger* (gi: 2351), *Aspergillus awamori* (gi: 454405), *Hypocrea jecorina* (gi: 261825113) and *Chaetomium thermophilum* (gi: 90654477). The conserved motifs are boxed. The putative catalytic residues are indicated with asterisks. The amino acid residues related to substrate binding and hydrolysis are indicted with pounds and arrows, respectively.

The deduced amino acid sequence of *gla15* shows the highest identity and similarity of 64.2% with a glucoamylase from *Talaromyces* (*Rasamsonia*) *emersonii* [15]. Deduced GLA15 contains two domains, a catalytic domain of GH15 and a carbohydrate binding domain (CBM) of family 20. Multiple sequence alignment revealed that deduced GLA15 has five conserved regions typical of fungal glucoamylases (Fig. 1). Structure analysis (data not shown) indicated that GLA15 is a typical glucoamylase, and Glu207 and Glu428 are putative catalytic residues, corresponding to the proton donor and acceptor, respectively. Five strictly conserved residues of GH15 related to substrate binding, including Tyr76, Trp80, Trp148, Arg333 and Tyr339, were also found in the catalytic module of GLA15 [28],[29].

### Expression and purification of GLA15

**Figure 2:**
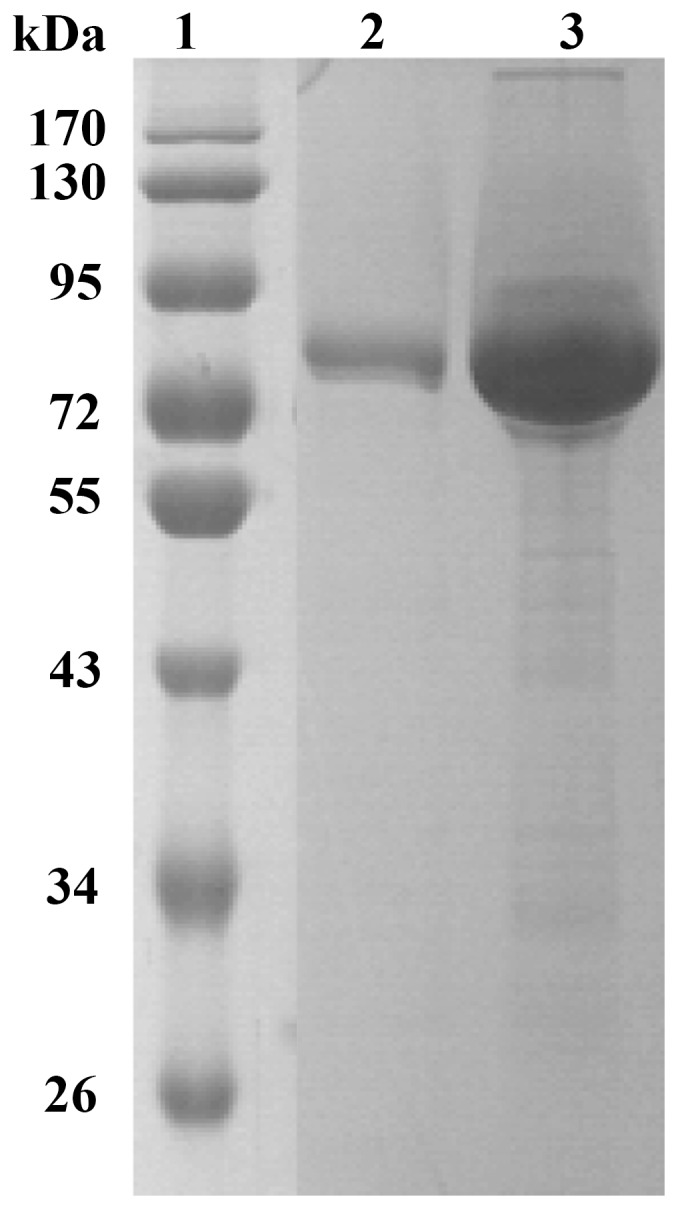
SDS-PAGE analysis of recombinant GLA15. *Lane 1*, the molecular mass standards; *lane 2*, the purified recombinant GLA15; *lane 3*, the crude recombinant GLA15.

The cDNA fragment of mature GLA15 without the signal peptide-coding sequence was amplified by PCR and cloned into the pPIC9 vector for heterologous expression in *P. pastoris* GS115. After 60 h growth in conical flasks at 30°C, the glucoamylase activity and protein content in the culture supernatant were determined to be 34.1 U/ml (pH 4.0 and 70°C for 10 min with soluble starch as the substrate) and 0.121 mg/ml, respectively. Recombinant GLA15 was purified to electrophoretic homogeneity with a yield of about 27.4%. SDS-PAGE analysis showed that the molecular mass of GLA15 was about 80 kDa (Fig. 2), which is higher than its theoretical molecular mass (63.3 kDa). Four *N*-glycosylation sites were predicted at positions Asn140, Asn199, Asn370 and Asn423 by NetNGlyc 1.0 server (http://www.cbs.dtu.dk/services/NetNGlyc/).

### Characterization of purified recombinant GLA15

**Figure 3:**
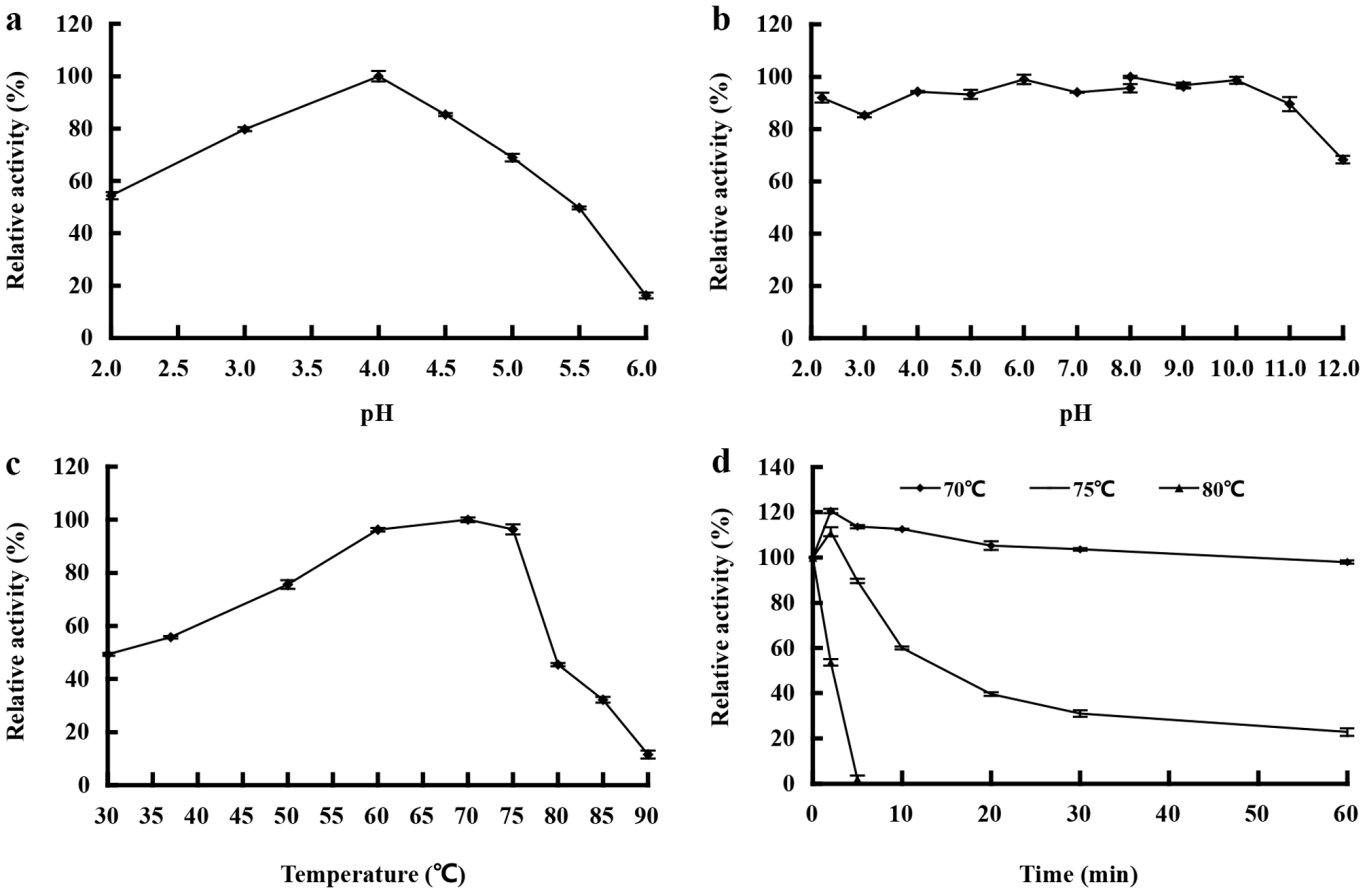
Characterization of purified recombinant GLA15. **a** Effect of pH on enzyme activity. **b** pH stability. **c** Effect of temperature on enzyme activity. **d** Thermostability assay. Each value in the panel represents the means ± SD (n = 3).

The pH optimum of GLA15 for the glucoamylase activity was pH 4.0 at 70°C, and more than 60% of the maximum activity was retained at pH 2.0–5.0 (Fig. 3a). GLA15 was highly stable over a broad pH range, remaining >85% of the activity after incubation at pH 2.2 to 11.0, 37°C for 1 h (Fig. 3b). The optimum temperature of GLA15 was 70–75°C (Fig. 3c). GLA15 was highly stable at temperatures up to 70°C (Fig. 3d).

The enzymatic activity of GLA15 was enhanced by most chemicals tested except for Mn^2+^, Ag^+^, Hg^2+^ and SDS at the concentration of 5 mM (Table 1). β-Mercaptoethanol at 5 mM even enhanced the enzyme activity by 82%. When increased the concentration to 10 mM, only Cu^2+^, Ca^2+^, Ag^+^, Hg^2+^, and SDS inhibited the enzyme.

**Table 1:** Effect of metal ions and chemical reagents on the activity of purified GLA15.

Chemicals	Relative activity (%)^a^		Chemicals	Relative activity (%)	
	5 mM	10 mM		5 mM	10 mM
None	100.0±0.7	100.0±1.4	Cr^3+^	112.9±1.3	125.1±0.3
Mg^2+^	142.2±0.9	135.5±1.6	Co^2+^	112.3±0.5	174.4±1. 7
Na^+^	140.4±0.7	111.6±1.3	Li^+^	111.3±1.0	132.0±1.5
Zn^2+^	140.1±0.4	128.6±0.5	K^+^	110.3±0.5	140.4±0.8
Cu^2+^	124.2±0.4	87.4±1.3	Mn^2+^	73.0±1.3	109.8±1. 6
Ca^2+^	116.7±0.3	83.7±0.5	Ag^+^	56.7±1.6	36.4±1.4
Ni^2+^	115.9±1.0	106.9±1.8	Hg^2+^	12.4±1.4	9.8±1.2
Fe^3+^	113.9±0.2	133.3±0.7	SDS	11.4±2.0	13.0±1.2
Pb^2+^	113.1±1.7	118.9±1.4	β-Mercaptoethanol	181.8±0.9	106.1±1.1

a Values represent the mean ± SD (n = 3) relative to the untreated control sample.

### Substrate specificity and kinetic values of GLA15

The substrate specificity of GLA15 was examined with various maltosaccharides as the substrate. As shown in Table 2, when defined the enzymatic activity towards soluble starch as 100%, GLA15 has the highest specific activity on amylopectin (117%), maltooligosaccharides (102%), soluble starch (defined as 100%) and amylose (97%), moderate on glycogen (73%) and dextran (64%), and low on raw starch (28%) and maltose (24%).

**Table 2:** Substrate specificity of purified recombinant GLA15.

Substrate	Specific activity (U/mg)	Relative activity (%)^a^
Amylopectin	2405.2±0.3	117
Maltooligosaccharides	2097.5±0.5	102
Souble starch	2051.5±0.7	100
Amylose	1984.9±1.1	97
Glycogen	1491.4±1.0	73
Dextran	1310.9±1.7	64
Raw starch	565.6±1.2	28
Maltose	498.0±1.5	24

a The enzymatic activity of GLA15 towards soluble starch is defined as 100%.

The kinetic analysis indicated that GLA15 has better binding affinity and higher catalytic efficiency to soluble starch than maltose (1.25 mg/ml vs. 4.85 mg/ml and 2100 ml/s/mg vs. 167.5 ml/s/mg, respectively).

### Cleavage mode and hydrolysis product analysis

GLA15 hydrolyzed maltooligosaccharides effectively as well as maltopolysacchardes. With maltooligosaccharides (G3–G7) as the substrates (Table S1), the enzyme was more efficient to hydrolyze oligosaccharides with longer chains than those short ones. After incubation at 60°C for 5 min, 49%, 80%, 83%, 100% and 100% of maltotriose, maltotetraose, maltopentose, maltohextose and maltoheptaose were hydrolyzed, respectively. When the incubation was prolonged to 120 min, all maltooligosaccharide substrates were completely degraded into glucose. The hydrolysis capacity of GLA15 against maltose was greater than that of maltotriose (68% vs. 49%, 5 min), but lower than that of other maltooligosaccharides (G4–G7). When used isomaltose as the substrate, 9% and 45% of isomaltose was hydrolyzed after 20 min and 120 min incubation, respectively. The results indicated that GLA15 has the ability to attack α-1,6-glycosidic linkages in isomaltose.

**Figure 4:**
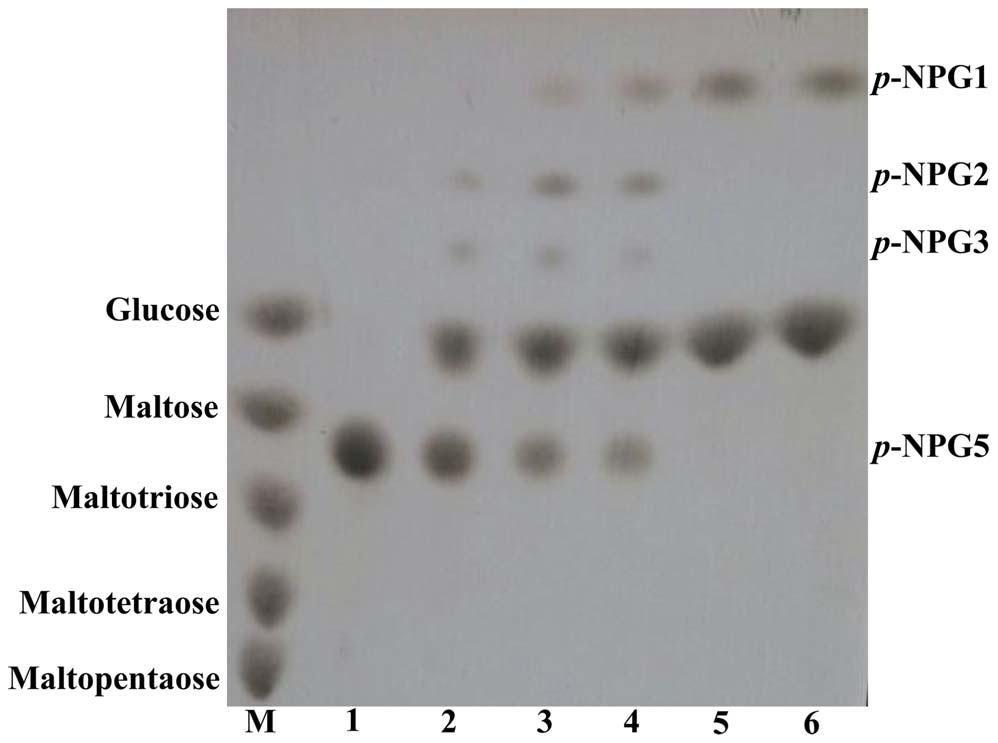
TLC analysis of the hydrolysis products of 4-nitrophenyl-α-d-maltopentaoside (*p*-NPG5) by GLA15. *Lane M*, the maltooligosaccharide standards (glucose to maltopentaose); *lanes 1*–*6*, the hydrolysis products of *p*-NPG5 after incubation with GLA15 for 0 min, 10 min, 30 min, 1 h, 3 h, and 10 h, respectively.

**Figure 5:**
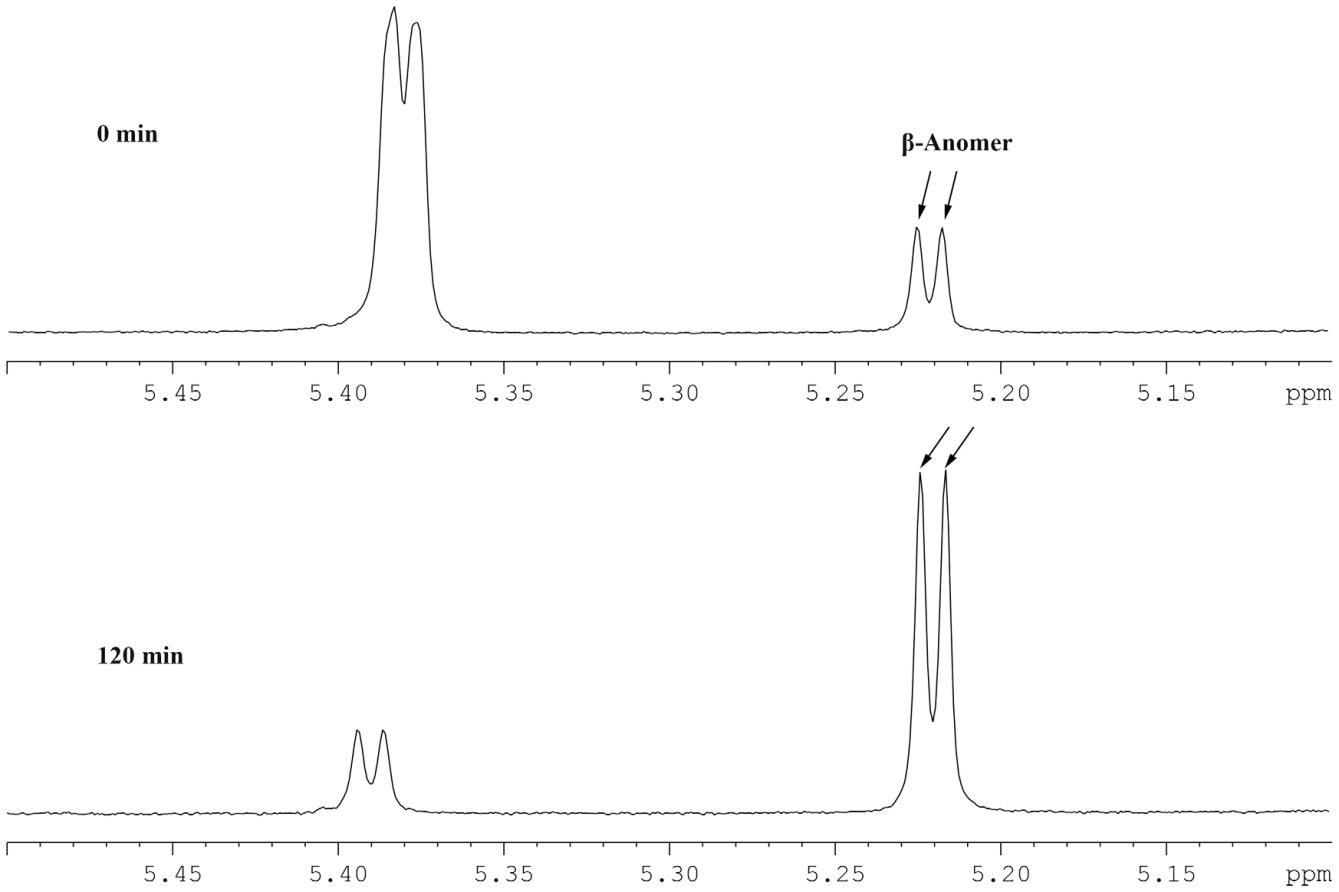
^1^H-NMR spectrum of maltotriose hydrolysates by GLA15. The arrows indicate the corresponding peaks of β-anomer.

TLC and ^1^H-NMR techniques were employed to determine the cleavage mode of GLA15 and glucose configuration, respectively. As shown in Fig. 4, *p*-NPG1–3 and glucose were observed in TLC plate when incubated *p*-NPG5 and GLA15 at 60°C for 10 min. *p*-NPG4 might be mixed with glucose and thus undetectable as reported on the glucoamylase of *Sulfolobus solfataricus* [12]. When the incubation was prolonged to 3 h, *p*-NPG5 was completely degraded into glucose and *p*-NPG1. The result indicated that GLA15 mainly attacked the α-1,4-glycosidic linkages from the non-reducing end to release glucose as the final products. ^1^H-NMR spectrum (Fig. 5) showed that the product of maltotriose was β-anomeric glucose (5.2 to 5.25 ppm), and its amount gradually increased as the reaction continued to 120 min. These results in combination indicated that GLA15 is a typical glucoamylase of GH15.

### Starch hydrolysis by enzyme combination and glucose reverse reaction

The hydrolysis abilities of GLA15 and commercial glucoamylase CGA towards gelatinized maize starch were compared. The highest saccharification efficiency, 96.14% for GLA15 and 87.37% for CGA, were observed at 6 h. When the incubation was prolonged to 8 h, the saccharification efficiency decreased by approximately 10%. The result suggested that GLA15 may catalyze the reverse reaction of glucose, a phenomenon popular in glucomylases [12],[30].

Prolonged co-incubation of GLA15 and 30% glucose resulted in the production of isomaltose (main product) and a little proportion of maltose (Table 3). The result confirmed that glucose reverse reaction occurred in the presence of GLA15, which probably contributed to the decreased saccharification rates when the incubation was prolonged. Enzyme dosages also had effects on reverse reactions. Low enzyme dosage (24 U/g glucose) required shorter incubation time (25 h) for glucose reversion than high enzyme dosage (48 and 72 U/g glucose, 50 h). The amounts of reverse products increased along with the increased enzyme amounts, and maintained constant levels after 50 h.

**Table 3:** The reversion reaction of glucose catalyzed by GLA15.^a^

Enzyme dosage (U/g glucose)	The concentrations of isomaltose/maltose (mg/l) at the following time (h)					
	0	0.5	5	25	50	150
24	0/0	0/0	44.0/0	133.4/11.1	138.0/19.9	114.1/83.2
48	0/0	7.8/0	62.0/0	154.4/0	223.7/14.2	178.5/109.5
72	0/0	11.5/0	76.6/0	154.5/0	309.4/18.1	242.4/125.8

a The main products of glucose reversion reaction are isomaltose and maltose.

## Discussion

At present, the glucoamylases from *A. niger* or closely related species are the most widely used in the saccharification stage of starch industry because of the beneficial properties for industrial bioprocesses such as high productivity and the ability to convert starch completely to glucose [31]. However, their pH and temperature optima (60–65°C, pH 4.0–4.5) and thermoliability require adjustment of the temperature and pH in the process of produce glucose. A thermostable glucoamylase that is highly active at the pH and temperature close to the liquefaction step (90–105°C and pH 5.5–6.0) can improve the starch conversion efficiency and save cost. Compared with its counterparts, the most important distinguished property of GLA15 is its excellent thermostability at 70°C. It retained 98% initial activity after incubation at 70°C for 1 h. By contrast, the commercial *A. niger* glucoamylase only retained 10% activity after incubation at 70°C for 30 min [5]; the *T. emersonii* glucoamylase only retained stable at 65°C [15]; the glucoamylase from *C. thermophilum* lost 20% activity after incubation at 70°C for 1 h [17]; and another *A. niger* glucoamylase with temperature optimum at 75°C was only stable at 40°C [30]. Even though the pH optimum of recombinant GLA15 was 4.5, it retained stable over a broader pH range (2.2–11.0) than glucoamylases from *A. niger* and *Aspergillus niveus* (pH 4.0–9.5) [32] and endophytic fungus EF6 (pH 4.0–7.0) [33]. The broad pH stability range and high-temperature thermostability of GLA15 can greatly increase the rate of saccharification reactions (or decreased amounts of enzyme required to give the same reaction rate as at 60°C), decrease microbial contamination of reaction vessels and decrease the viscosity of reaction syrups. However, the enzyme activity of GLA15 greatly decreased at pH 6.0 and above. To make it suitable and economic for industrial application, we will employ molecular/protein engineering techniques to improve GLA15 activity at neutral pH in future studies.

Glucoamylases are biotechnologically very important as they are used industrially in large amounts. Researchers have identified and characterized various glucoamylases from archaea [12],[13] and bacteria [9],[10],[11]. Even though the temperature and pH optimum (≧70°C, pH 5.0–6.0) of these glucoamylases are very close to the conditions of liquefaction, they have many disadvantages, such as low enzyme production yields, slow microbial growth and poor heterogeneous expression. In the present study, thermostable GLA15 with a broad pH range was produced in *P. pastoris* with a high yield of 34.1 U/ml, significantly higher than that of glucoamylases from *C. thermosaccharolyticum* (1.42 U/ml) [10], *Sulfolobus solfataricus* (2.08 U/ml) [12] and *Thermomyces lanuginosus* (7.3 U/ml) [16]. The high yield makes recombinant GLA15 meet with the market requirement.

Industrial saccharification typically results in 96% maximum conversion to glucose [34]. The GLA15 potential for application in the starch industry was assessed. Our results showed that GAL15 had a saccharification efficiency of 96.14%, higher than the commercial *A. niger* glucoamylase (87.37%). This high conversion rate might be ascribed to the relatively broader substrate specificity, including maltooligosaccharides, polysaccharides, and even raw starch. Most fungal glucoamylases have preference for large molecules, such as starch, amylopectin and glycogen, rather than small maltooligosaccharides [11], and the catalytic efficiency towards maltooligosaccharides is increased with higher degree of polymerization [10]. GLA15 shows similar substrate preference. The wide substrate specificity of GLA15 is much valuable since the liquefaction reaction catalyzed by amylase produces oligosaccharides of different sizes [35]. On the other hand, its ability to hydrolyze maltose is similar to that of α-glucosidase, which converts maltodextrins, especially maltose, into glucose. The only difference is that GLA15 liberated β-d-glucose from the non-reducing end of the substrate, while α-glucosidase releases α-d-glucose [12]. The mechanism of GLA15 for significant hydrolysis ability towards maltooligosaccharides and high glucose yield is also primarily undermined. Previous mutation studies have revealed some key residues related to substrate hydrolysis. For example, Ser30 in *Aspergillus awamori* glucoamylase, equivalent to Pro58 in GLA15, is another key residue related to substrate binding. Its substitution with Pro not only reduced the conformational unfolding, but also favored the α-1,4 binding mode at the active site [36]. Moreover, site-directed mutation of G137A (corresponding to Ala165 in GLA15) in *A. awamori* glucoamylase changed the conformation around the catalytic cavity by making helix-4 more stable, which disfavored its binding to α-1,6-linked substrate and resulted in higher glucose yields [36]. Furthermore, the catalytic efficiency and glucose yield of *A. awamori* glucoamylase for isomaltose hydrolysis significantly increased when substituted Tyr175 (Phe203 in GLA) with Phe [36]. Based on sequence alignment (Fig. 1), these hydrolysis-favorable residues were also identified in GLA15, which may contribute to the broad substrate specificity and high saccharification efficiency in combination.

To be economical, high concentrations of starch (up to 30%) are generally used in the industrial process, which may result in high concentrations of glucose and formation of glucose reversion products [12]. The main reversion product of GLA15 was isomaltose, and maltose didn't appear until 50 h. Considering the preference for α-1,4-glycosidic to α-1,6-glycosidic linkages (Table S1) and the weak ability to hydrolyze glucose reversion products of GLA15, strict control of the glucose concentration and saccharification time is feasible to optimize the starch processing. In summary, GLA15 is an excellent candidate for application in industrial starch processing, not only because of its better thermostability (70°C) and stability over a broad pH range (2.2–11.0), but also because of the higher starch hydrolysis capacity (96.14%) and high expression yield (34.1 U/ml). Its activity at neutral pH will be improved by molecular/protein engineering techniques.

## Supporting Information

Table S1 **The product compositions of maltooligosaccharides and isomaltose hydrolyzed by GLA15.**
(DOC)
