# Supplementary material for: A Thermostable Glucoamylase from Bispora sp. MEY-1 with Stability over a Broad pH Range and Significant Starch Hydrolysis Capacity
Source: PLoS One. 2014 Nov 21;9(11):e113581. doi: 10.1371/journal.pone.0113581 (PMC4240638; doi:10.1371/journal.pone.0113581)
Supplement: Table S1 — The product compositions of maltooligosaccharides and isomaltose hydrolyzed by GLA15. (DOC) [file pone.0113581.s001.doc]

**PLoS ONE**

**A thermostable glucoamylase from *Bispora* sp. MEY-1 with stability over a broad pH range and significant starch hydrolysis capacity**

Huifang Hua^1#^, Huiying Luo^1#^, Yingguo Bai^1^, Kun Wang^1^, Canfang Niu^1^, Huoqing Huang^1^, Pengjun Shi^1^, Caihong Wang^1^, Peilong Yang^1,2^, Bin Yao^1*^

**1** Key Laboratory for Feed Biotechnology of the Ministry of Agriculture, Feed Research Institute, Chinese Academy of Agricultural Sciences, Beijing 100081, P. R. China, **2** CAAS-ICRAF Joint Laboratory on Agroforestry and Sustainable Animal Husbandry, Beijing 100193, P. R. China

^#^ Huifang Hua and Huiying Luo contributed equally to this article.

^*^ Corresponding author. Address: Key Laboratory for Feed Biotechnology of the Ministry of Agriculture, Feed Research Institute, Chinese Academy of Agricultural Sciences, No. 12 Zhongguancun South Street, Beijing 100081, P. R. China. Tel.: +86 10 82106053; fax: +86 10 82106054. e-mail: [binyao@caas.cn](mailto:binyao@caas.cn), yaobin@caas.cn

**Table S1**

The product compositions of maltooligosaccharides and isomaltose hydrolyzed by GLA15.

| Oligosaccharides | Degree of polymerization | Product percentages at the time points ^a^ | | | |
| --- | --- | --- | --- | --- | --- |
|  |  | 1 | 2 | 3 | 4 |
| Maltose | 2 | 100 | 32 | 19 | 1 |
|  | 1 | 0 | 68 | 81 | 99 |
| Maltotriose | 3 | 100 | 51 | 18 | 0 |
|  | 2 | 0 | 24 | 38 | 1 |
|  | 1 | 0 | 25 | 44 | 99 |
| Maltotetraose | 4 | 100 | 20 | 0 | 0 |
|  | 3 | 0 | 24 | 12 | 0 |
|  | 2 | 0 | 12 | 26 | 1 |
|  | 1 | 0 | 44 | 62 | 99 |
| Maltopentaose | 5 | 100 | 17 | 0 | 0 |
|  | 4 | 0 | 3 | 2 | 0 |
|  | 3 | 0 | 25 | 17 | 0 |
|  | 2 | 0 | 0 | 14 | 0 |
|  | 1 | 0 | 55 | 67 | 100 |
| Maltohexaose | 6 | 100 | 0 | 0 | 0 |
|  | 5 | 0 | 18 | 0 | 0 |
|  | 4 | 0 | 13 | 3 | 0 |
|  | 3 | 0 | 12 | 16 | 0 |
|  | 2 | 0 | 0 | 8 | 0 |
|  | 1 | 0 | 57 | 73 | 100 |
| Maltoheptaose | 7 | 100 | 0 | 0 | 0 |
|  | 6 | 0 | 0 | 0 | 0 |
|  | 5 | 0 | 15 | 0 | 0 |
|  | 4 | 0 | 13 | 5 | 0 |
|  | 3 | 0 | 6 | 12 | 0 |
|  | 2 | 0 | 0 | 6 | 0 |
|  | 1 | 0 | 66 | 77 | 100 |
| Isomaltose | 2 | 100 | 91 | 61 | 55 |
|  | 1 | 0 | 9 | 39 | 45 |

^a^ Hydrolysis product compositions were determined at 0, 20, 60, and 120 min for isomaltose and 0, 5, 10, and 120 min for other substrates, respectively.
